# Structural and compositional study of precipitates in under-aged Cu-added Al-Mg-Si alloy

**DOI:** 10.1038/s41598-018-35134-8

**Published:** 2018-11-09

**Authors:** Takuya Maeda, Kenji Kaneko, Takuya Namba, Yuki Koshino, Yukio Sato, Ryo Teranishi, Yasuhiro Aruga

**Affiliations:** 1 Department of Materials Science and Engineering, Kyushu University, 744, Motooka, Nishi, Fukuoka 819-0395 Japan; 2 Materials Research Laboratory, Kobe Steel, Ltd., 1-5-5, Takatsukadai, Nishi, Kobe 651-2271 Japan

## Abstract

Atomic scale characterization of fine precipitates in an under-aged Cu added Al-Mg-Si alloy was carried out by combination of atomically-resolved annular dark-field scanning transmission electron microscopy and energy dispersive X-ray spectroscopy. Two types of precipitates were observed in the alloy. In the case of ordered β” precipitates, β” was proposed as Mg_5-x_Al_2+x_Si_4_ (x ≈ 1) with solute Cu atoms replacing Al site of β” precipitate. In the case of disordered precipitates, the precipitates were found to consist of β” sub-unit cells, three-fold symmetric structure without Cu atoms, Cu containing structures termed as “Cu sub-unit cluster”, and Q’ sub-unit cells. Among these structures, the morphologies of three-fold symmetric structure without Cu atoms, Cu sub-unit cluster, and Q’ sub-unit cell were almost the same, so that these structures should be the clusters of Q’ phase. Since the areal density, length and diameter of precipitates were almost equal between Cu free Al-Mg-Si alloy and Cu added Al-Mg-Si alloy, the increase of hardness by Cu addition should be due to the precipitation of Cu related precipitates, such as Cu sub-unit clusters and Q’ sub-unit cells.

## Introduction

Precipitation hardening is one of the most effective routes to improve the strength of age-hardening aluminum alloys due to the movement of dislocations being hampered by the precipitates^1–9^. In general, age-hardenable Al-Mg-Si alloys show a combination of superior mechanical properties, such as high strength, high formability, high weldability and high corrosion resistance, so that they are commonly used in construction materials and automotive components^3–6,10–13^. A large number of research studies has already been carried out to reveal the precipitation sequences and to understand the correlation between the strength and microstructures, in order to control the size, dispersion, and microstructures of precipitates for further improvement of mechanical properties^14–24^. The precipitation sequences in Al-Mg-Si alloys are accepted as^19–28^:

$$\begin{array}{rcl}{\rm{supersaturated}}\,{\rm{solid}}\,{\rm{solution}}\,({\rm{SSSS}}) & \to  & {\rm{atomic}}\,{\rm{clusters}}\to {\rm{G}}{\rm{.P}}.\,{\rm{zones}}\\  & \to  & {\rm{metastable}}\,{\rm{\beta }}\mbox{''}\,{\rm{phase}}\\  & \to  & {\rm{metastable}}\,{\rm{\beta }}\mbox{'},\,{\rm{U1}},\,{\rm{U2}},\,{\rm{{\rm B}}}{\rm{\mbox{'}}}\,{\rm{phases}}\\  & \to  & {\rm{stable}}\,{\rm{\beta }}\,{\rm{phase}}.\end{array}$$

The peak strength of the Al-Mg-Si alloy is known to be caused by the dispersion of β” phase, which can be changed by addition of Mg and/or Si, as well as other elements^6,15–17,29^.

In particular, addition of Cu to the Al-Mg-Si alloys shows large enhancement of the age-hardening kinetics and improvement of the peak hardness, and have been widely applicable for the industrial applications owing to their good mechanical properties compared with the one without Cu^15–17^. The cause of these phenomena have been considered as the change of precipitation sequences as^29–35^:

$$\begin{array}{c}{\rm{SSSS}}\to {\rm{atomic}}\,{\rm{clusters}}\to {\rm{G}}{\rm{.P}}.\,{\rm{zones}}\to {\rm{metastable}}\,{\rm{\beta }}\mbox{'}\mbox{'},\,{\rm{L}},\,{\rm{C}},\,\text{QP},\,{\rm{QC}}\,{\rm{phases}}\\ \to {\rm{metastable}}\,{\rm{\beta }}\mbox{'},\,{\rm{Q}}\mbox{'}\,{\rm{phases}}\to {\rm{stable}}\,{\rm{Q}}\,{\rm{phase}}\end{array}$$

Every single characteristics of precipitates, not only size and dispersion, but also structures and compositions of the precipitates play important roles for the mechanical properties. It is therefore various experimental techniques, mainly microscopies, spectroscopies and diffractometries, have been carried out to reveal the compositions and microstructures of precipitates in detail. Table 1 shows an overview of the reported precipitates in Al-Mg-Si-(Cu) alloys.

**Table 1:** Overview of the reported precipitates in the Al-Mg-Si-(Cu) alloy.

Precipitates	Morphology	Composition	Lattice structure (nm)	Ref.
G.P. zone		Mg_2+x_Al_7-x-y_Mg_2+y_ (1 < x + y < 3)	Monoclinic *a* = 1.48, *b* = 0.405, *c* = 0.674, *β* = 105.3°	^27^
β”	Needle	Mg_5_Si_6_ Al_2_Mg_5_Si_4_ Al_3_Mg_4_Si_4_	Monoclinic *a* = 1.516, *b* = 0.405, *c* = 0.674, *β* = 105.3°	^20, 21, 36^
U1	Needle	MgAl_2_Si_2_	Trigonal *a* = *b* = 0.405, *c* = 0.674, *γ* = 120°	^26^
U2	Needle	MgAlSi	Orthohombic *a* = 0.675, *b* = 0.405, *c* = 0.794	^23^
B’	Lath	Mg_9_Al_3_Si_7_	Hexagonal *a* = 1.04, *c* = 0.405, *γ* = 120°	^19, 33^
β’	Needle	Mg_1.8_Si	Hexagonal *a* = 0.715, *c* = 0.405, *γ* = 120°	^22^
QP	Needle	Unknown	Hexagonal *a* = 0.393, *c* = 0.405	^35^
QC	Needle	Unknown	Hexagonal *a* = 0.670, *c* = 0.405	^35^
C	Plate	Unknown	Monoclinic *a* = 1.032, *b* = 0.81, *c* = 0.405, *γ* = 101°	^34^
L	Needle	Unknown	Unknown	^31^
Q’	Needle	Al_3_Cu_2_Mg_9_Si_7_ Al_6_Mg_6_Si_7_Cu_2_	Hexagonal *a* = 1.032, *c* = 0.405, *γ* = 120°	^31, 38– 40^
β	Plate	Mg_2_Si	Cubic *a* = 0.635	^28^
Q	Needle	Al_3_Cu_2_Mg_9_Si_7_	Hexagonal *a* = 1.039, *c* = 0.402, *γ* = 120°	^39^

The structures and the compositions of β” and Q’ precipitates have been studied intensively in past, and those of β” precipitates have been suggested as monoclinic with Mg_5_Si_6_^21,36^, Mg_5_Al_2_Si_4_^37^, or Al_3_Mg_4_Si_4_^20^ stoichiometry, and those of Q’ as hexagonal with Al_3_Mg_9_Si_7_Cu_2_^31,38,39^ or Al_6_Mg_6_Si_7_Cu_2_^40^ stoichiometry. Wenner *et al*. carried out atomically-resolved annular dark-filed scanning transmission electron microscopy (ADF-STEM) with energy dispersive X-ray spectroscopy (EDS) to determine the composition of β” precipitates as Mg_5_Al_2_Si_4_, and Q’ as Al_6_Mg_6_Si_7_Cu_2_ from over-aged Al-Mg-Si alloy^40^, as decisive experimental evidence. The compositions of these reported over-aged precipitates are expected to be different from under-aged ones. Both compositions and structures of over-aged precipitates should be dependent on the earlier stage of the precipitation, so that the microstructural characterization of under-aged precipitates are necessary for understanding of the transformation of precipitates and their sequences. Moreover, determination of substitutional sites of Cu atoms in β” precipitate is also important for clarification of transformation mechanisms of β” into the other precipitates.

In this work, atomically-resolved ADF-STEM and EDS column mapping were carried out for the characterization of under-aged fine precipitates in both Cu free and Cu added Al-Mg-Si alloys. We present the microstructures and compositions of fine precipitates and discuss the effect of Cu addition for the precipitation sequences in under-aged Al-Mg-Si alloys.

## Experimental Method

### Sample

Compositions of two Al-Mg-Si alloys are listed in Table 2. Both alloys were solutionized at 823 K for 1.8 ks, followed by water quenching, and aged isothermally at 453 K for different periods. The variation of hardness with ageing period of both alloys were measured by a Vickers micro hardness tester (MVK-H1, Akashi Co., Japan). Average hardness of both alloys were measured with 0.5 kgf load for a holding time of 10 s from 5 times of measurements.

**Table 2:** Composition of the Al-Mg-Si alloys observed in this experiment. (mass%).

	Mg	Si	Cu	Fe	Al
4M10S	0.431	1.049	0.001	0.122	Bal.
6016	0.432	1.007	0.172	0.164	Bal.

TEM specimens were prepared by electro polishing machine (Model 110 Automatic Twin-Jet Electropolisher, E.A. Fischione Instruments, U.S.) used with an electrolyte solution composed of 10 vol% of HClO_4_ and 90 vol% of C_2_H_5_OH. Electro polishing was performed below 283 K by liquid nitrogen at a few tens of mA under 20 V.

Clusters (or precipitates) of under-aged alloys for 1.8 ks were examined by combination of atomically resolved HAADF-STEM and EDS in order to analyze the precipitation sequences of under-aged precipitates. Both transmission electron microscopy (TEM) and atomically-resolved annular dark-field STEM (ADF-STEM) characterizations were carried out by JEM-ARM200CF (JEOL, Japan) equipped with a cold field emission gun and a spherical aberration corrector (CEOS, Germany) for the electron probe. TEM was operated at 120 kV to minimize the irradiation damage over the sample during the observation. The semi-angle of probe forming aperture was set between 28 and 33 mrad for the STEM observation. Since precipitates in 4M10S sample should only be composed of light elements, Mg, Al, Si, so that the middle-angle ADF-STEM (MAADF-STEM) with a collection semi-angle of 45–180 mrad was used for the acquisition of the images. On the other hand, high-angle ADF-STEM (HAADF-STEM) with a collection semi-angle of 90–370 mrad was used for the acquisition of images dominated by atomic number contrast (Z contrast) from 6016 sample. In order to minimize the specimen drift and to improve the image quality, series of atomically-resolved ADF-STEM images, as a size of pixels of 512 × 512, were acquired with a scanning pixel dwell time of 3 μs. From each sample, 42 to 70 STEM images were obtained, then the image series were overlayed by a non-rigid registration methods using SmartAlign software (HREM research, Japan)^41^.

Column mapping was carried out with a combination of energy dispersive X-ray spectroscopy (EDS) and STEM. The acceleration voltage was maintained at 60 kV in order to minimize the irradiation damage. X-ray spectrum were detected by silicon drift detector with collection solid angle of 2.0 sr. The column maps were acquired as a size of pixels of 256 × 256 with a frame time of 3.0 s, and iterating of the scan.

## Results

### Dispersion of precipitates and hardness of the alloys

**Figure 1:**
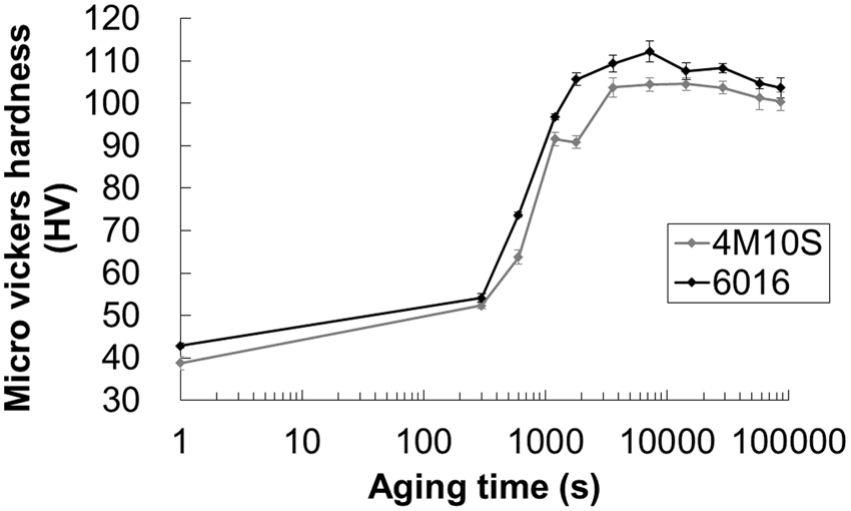
The variation of hardness with ageing period of 4M10S and 6016 alloys aged isothermally at 453 K.

Figure 1 shows the variation of hardness with ageing period of both 4M10S and 6016 alloys. The hardness of 6016 alloy was 42.8 ± 0.8 HV, which was found slightly harder than that of 4M10S alloy of 38.8 ± 0.3 HV at the shorter period, probably due to the solid solutioning of Cu. The hardness of both alloys became similar to each other at around 300 s, which suggested that the amount of solid solution strengthening by Cu atoms became small due to the precipitation in 6016 alloy and the amount of precipitation strengthening became equal between 4M10S and 6016 alloy. The hardness of both alloys became increased dramatically and reached the peak hardness at 7.2 ks, 112.2 ± 0.8 HV and 104.5 ± 1.2 HV for 6016 alloy and 4M10S alloy, respectively. The hardness of 6016 alloy was found harder than that of 4M10S alloy with further ageing time, which supports the hardness increase by the addition of Cu in Al-Mg-Si alloy, as reported previously^15,16^.

**Figure 2:**
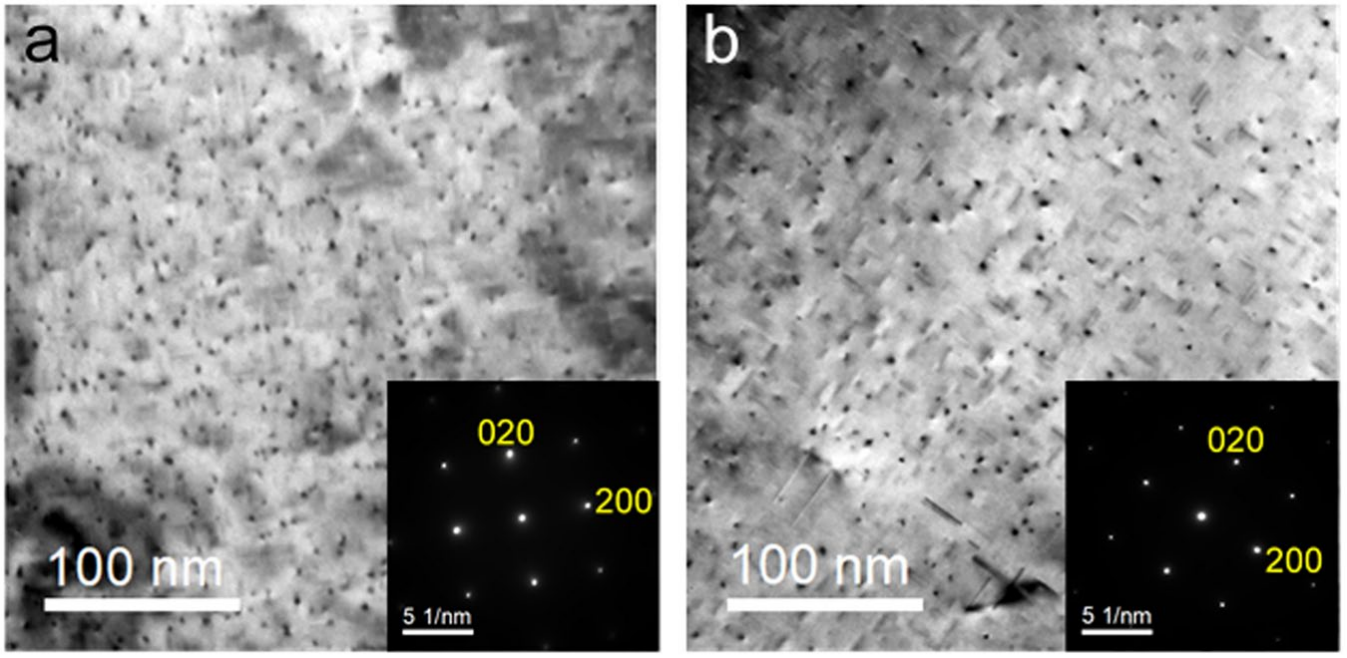
BF-TEM images of the precipitates in the Al-Mg-Si alloys and its SAED patterns. (**a**) 4M10S aged for 1.8 ks. (**b**) 6016 aged for 1.8 ks.

Figure 2(a,b) show BF-TEM images of uniformly-dispersed needle-shaped precipitates growing parallel to <100> matrix directions from under-aged 4M10S and 6016 alloys, respectively. Typical strain contrasts were also seen around the precipitates, split by a line without contrast parallel to the growth direction of the precipitate. Microstructures of precipitates of 4M10S and 6016 alloys aged for 1.8 ks are summarized in Table 3, and in particular, the length, diameter, and areal density of precipitates were almost the same for both alloys. The hardness of 6016 alloy was found harder than that of 4M10S alloy, which suggests that Cu additions in Al-Mg-Si alloy might have changed the structures of precipitates and resulted the increase of hardness.

**Table 3:** Number of the density, and the size of the precipitates in the both alloys isothermal aged for 1.8 ks at 453 K.

	4M10S	6016
Number of the density (m^−2^)	3.95 × 10^15^	3.85 × 10^15^
Length of the precipitates (nm)	9.79	9.17
Diameter of the precipitates (nm)	2.89	2.83

### Characterization of the precipitates in under-aged alloys

**Figure 3:**
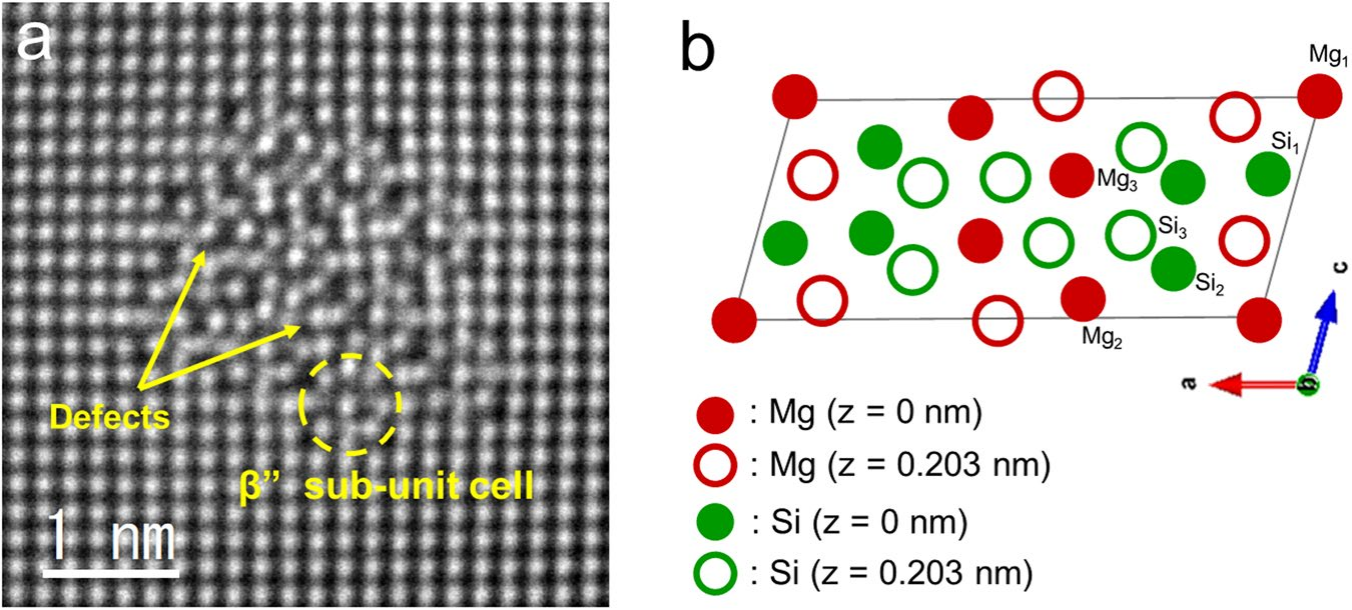
(**a**) Atomically-resolved MAADF-STEM images of the β” precipitate in under-aged 4M10S. (**b**) Schematic drawing of β” unit cell proposed as Mg_5_Si_6_.

Figure 3(a) shows a cross-sectional view of β” precipitate in under-aged 4M10S alloy by atomically-resolved MAADF-STEM, where one of the β” sub-unit cell is indicated by a dotted-line circle. Some defects were also present at the vicinity of β” sub-unit cells as indicated by arrows, which makes the distortion of β” unit cell, as schematically drawn in Fig. 3(b). Presence of disordered β” precipitates has also been reported in the case of an Al-Mg-Si-Cu alloy^30,42^, due to the precipitation of C, QP, QC, and Q’ unit cells at the β” region.

**Figure 4:**
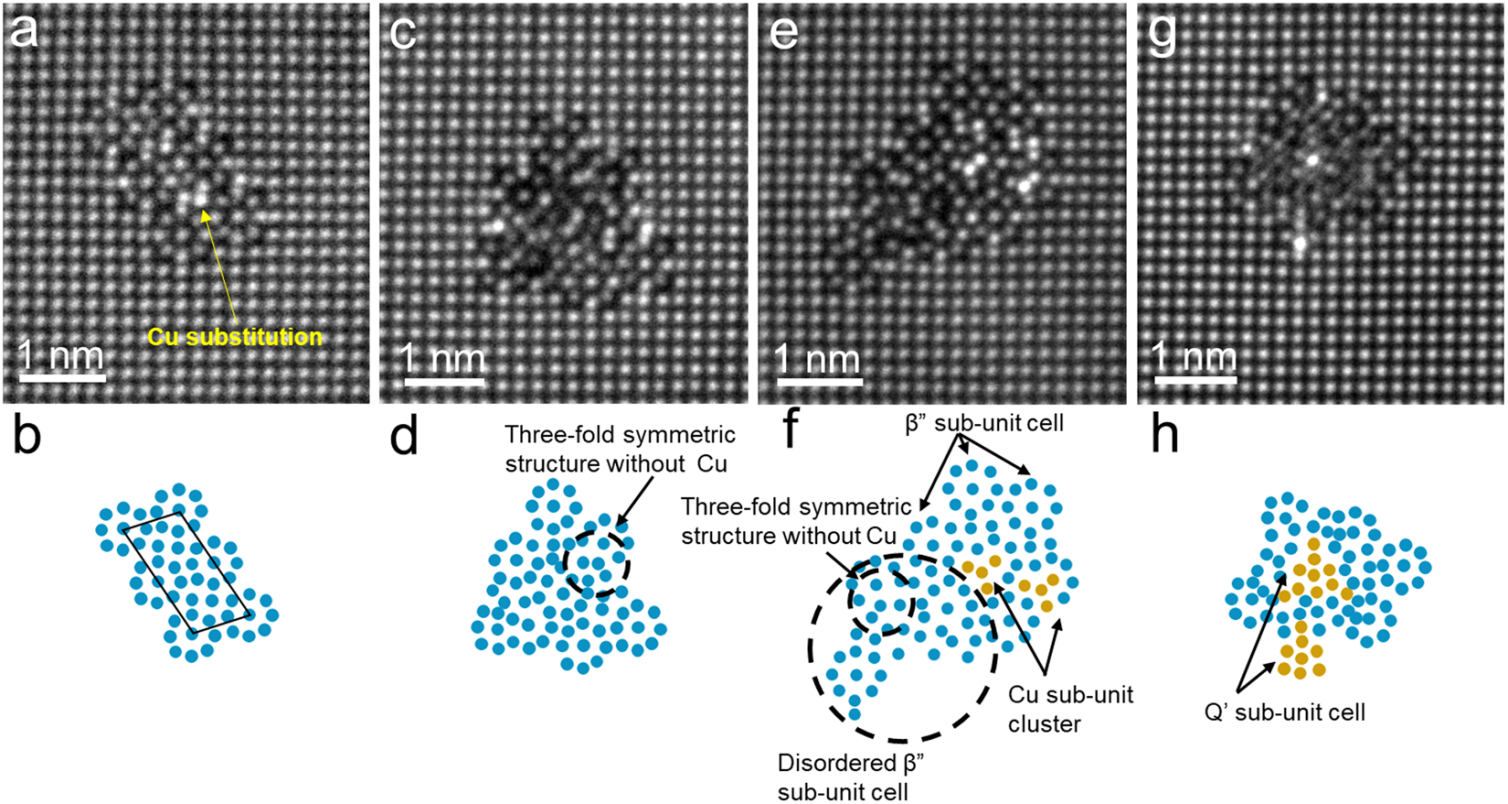
Atomically-resolved HAADF-STEM images of the precipitates in under-aged 6016 alloy. (**a**,**b**) shows β” precipitate consisted of unit cell and its schematic drawing. (**c**,**d**) shows disordered β” precipitate and its schematic drawing. (**e**,**f**) shows Cu containing sub-unit structures mixed with ordered and disordered β” sub-unit cells and its schematic drawing. (**g**,**h**) shows the sub-unit cells of Q’ phase surrounded by β” sub-unit cells and its schematic drawing.

Fig. 4 show four representative sets of images of β” precipitates observed by atomically-resolved HAADF-STEM with its schematic diagrams for under-aged 6016 alloy. Both ordered and disordered β” precipitates were commonly observed, as shown in Fig. 4(a–d), respectively. In Fig. 4(a,b), β” sub-unit cells forms parallelogram of a unit cell, which consist of 6 sub-unit cells with a few bright atomic columns, indicated by yellow arrow in Fig. 4(a). Atomic columns with bright contrast in the ordered β” precipitate is caused by the substitution of Cu atoms of Al, Mg or Si atoms in the β” precipitate.

In the case of disordered β” precipitate shown in Fig. 4(c), three-fold symmetric structure was found even without Cu atom (schematically drawn in Fig. 4(d)), which is very similar to the Cu containing structure, as shown in Fig. 4(e,f).

In Fig. 4(e), a Cu containing structure with three-fold symmetry were found in β” precipitate, as reported by Ding *et al*.^42^, which was termed as “Cu sub-unit cluster”. Some of β” sub-unit cells were found disordered in the β” precipitate, probably caused by the presence of Cu sub-unit clusters.

Although metastable Q’ phase is known to appear at the later stage of ageing, the Q’ sub-unit cells seemed coexisted already with β” sub-unit cells in under-aging period sample, as shown in Fig. 4(g), where the presence of Q’ sub-unit cells/precipitates were recognized by EDS column mapping, as the structure of hexagonal with Al_3_Mg_9_Si_2_Cu_7_ (shown in Supplement Fig. 1). In particular, one Q’ sub-unit cell was found surrounded by disordered and ordered β” sub-unit cells, and another Q’ sub-unit cell was also present at the vicinity of it, as schematically drawn in Fig. 4(h). One of the Q’ sub-unit cell shows good coherence with the matrix, and the interface between the sub-unit cell and the matrix is uncertain, probably due to the sub-unit cell being halfway stage of the precipitation.

### Column mapping of β” precipitate in 6016 alloy

**Figure 5:**
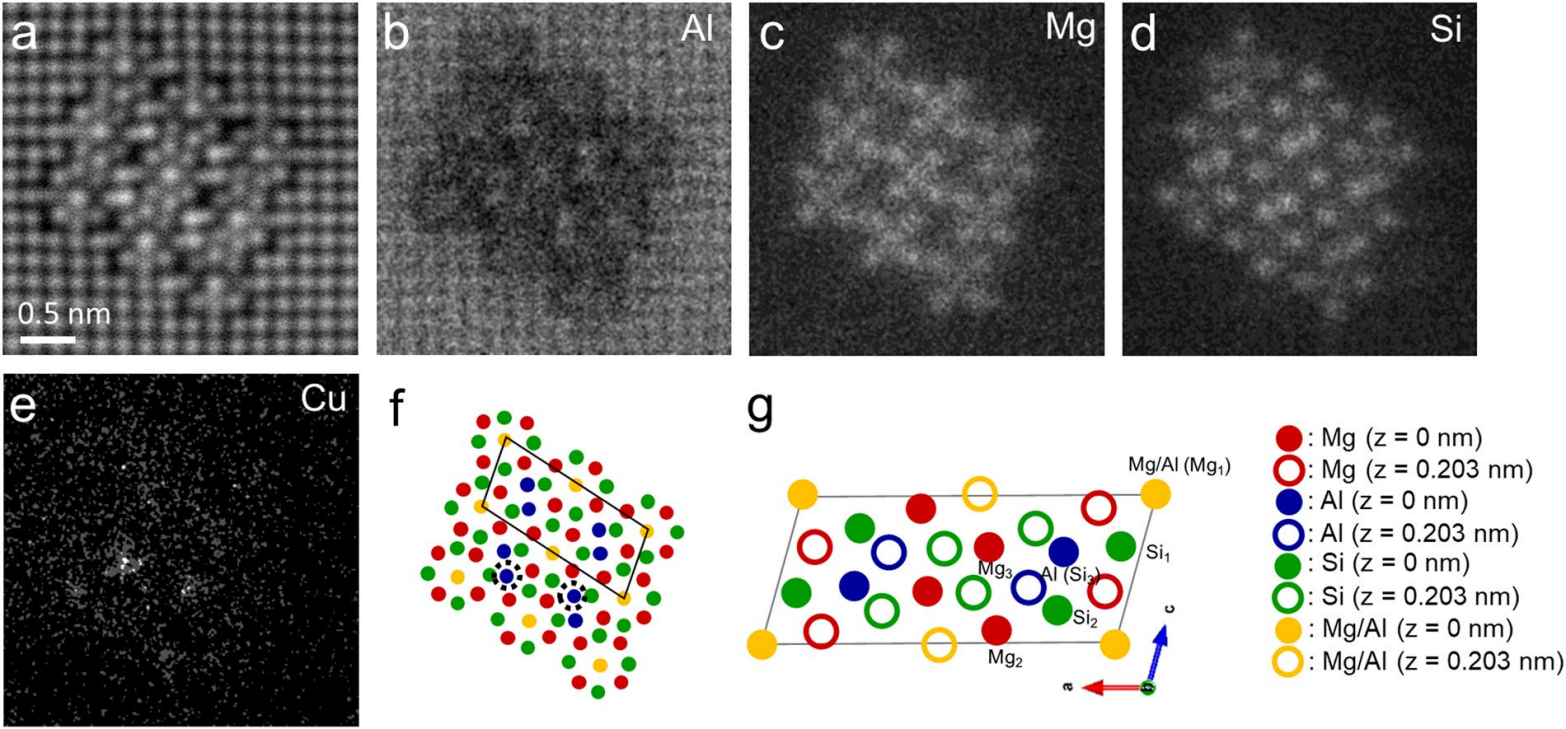
Atomically-resolved HAADF-STEM image and EDS maps of the β” phase in under-aged 6016 alloy. (**a**) shows HAADF-STEM image. (**b**–**e**) shows chemical map of Al, Mg, Si, and Cu, respectively. (**f**) shows schematic drawing of proposed β” structure from the EDS results. Dotted line circles indicate substitutional site of Cu atoms. (**g**) shows schematic drawing of suggested β” unit cell.

Figure 5 shows a set of HAADF-STEM image and EDS atomic column maps of β” precipitate in under-aged 6016 alloy, with its schematic diagram. As can be seen in Fig. 5(a–e), β” precipitate was found consisting of two β” unit cells and the structure was found consisting of Mg, Al, Si, and slight amount of Cu, as schematically drawn in Fig. 5(f). In past, the structure and composition of β” were proposed as monoclinic with Mg_5_Si_6_^21^ or monoclinic with Mg_5_Al_2_Si_4_^37^, as schematically drawn in Fig. 3(b). Furthermore, EDS column maps strongly suggests the presence of Al atoms at Si_3_ and Mg_1_ columns site, where Mg_1_ and Si_3_ sites are indicated in schematic drawing in Fig. 5(g). In Si_3_ columns, none of Si atom could be seen, so that Si atoms seemed substituted with Al atoms. On the other hand, Mg atoms were still present in Mg_1_ columns, so that Mg_1_ columns consisted of both Mg and Al atoms. Therefore, the composition of β” precipitate can now be proposed as Mg_5-x_Al_2+x_Si_4_, as schematically drawn in Fig. 5(f,g), probably very much similar to the structure of AlMg_4_Al_2_Si_4_ proposed by Hasting *et al*.^37^. Furthermore, the locations of substitutional Al atoms, which are Mg_1_ and Si_3_ columns, are also well matched with the one calculated via first principles calculations by Hasting *et al*.^37^.

Substitutional Cu atoms were also observed in the β” precipitate, replacing Al sites (Si_3_ columns) as shown in Fig. 5(e), which clarified that Cu atoms can substitute with Al atom as a solute atom, not only in the Al matrix but also in the β” precipitate.

## Discussion

Cayron *et al*. proposed the structure of metastable QC, QP, B’ (Q’) and stable Q as well as the precipitation sequences as^35,43^:

$${\rm{QP}}\to {\rm{QC}}\to {\rm{B}}\mbox{'},\,(Q\mbox{'})\to {\rm{stable}}\,{\rm{Q}}$$

This precipitation sequence of each phase could be explained by the fact that the phases are based on a common hexagonal latent lattice (QP lattice). Recently, more precise sequence of Cu added Al-Mg-Si alloy were proposed by Ding *et al*.^42^, where there are several transformation routes to be the stable Q phase via metastable phases as follows:

1. Cu atoms entered in β” evolve to Cu sub-unit clusters and the mixed sub-unit precipitates are formed.
2. The mixed sub-unit precipitates transform into the precipitate containing Q’ unit cell (referred to as disordered QP1) and the precipitate containing C unit cell (referred to as disordered QP2).
3. The disordered QP1 transforms to stable Q phase via Q’ phase, and the disordered QP2 transforms to Q’ or stable Q phase via formation of Cu sub-unit clusters, ordered QP lattice, and ordered QC lattice.

The presence of solute Cu atoms in β” precipitate was recognized qualitatively, as shown in Figs 4(a) and 5(e), which were only present in Al column (Si_3_ column). It is considered that these solute Cu atoms evolve to Cu sub-unit clusters, as proposed by Ding *et al*.^42^. Moreover, three-fold symmetric structure without Cu atoms is formed due to the formation of disordered β” (shown in Fig. 4(c,d)), which is also considered to transform into the Cu sub-unit cluster. The Cu sub-unit cluster then transforms into Q’ sub-unit cell in disordered β” precipitate, as shown in Fig. 4(g,h). Furthermore, Q’ sub-unit cell with good coherence with Al matrix was also observed, as shown in Fig. 4(g,h), so that Q’ sub-unit cell can also be formed directly at the interface of the disordered precipitate without transformation from the Cu sub-unit cluster. In addition, the disordered Q’ precipitate containing Q’ unit cell was observed in the sample aged for 1.8 ks, as shown in Supplement Fig. 1, which suggests that Q’ precipitates are also present as a dominant precipitate at under-aging period. Thus, the precipitation sequences in Al-Mg-Si-Cu alloy can be proposed as:

1. Transformation route: β” including solute Cu atoms, disordered β” with three-symmetric structure → Cu sub-unit cluster → Q’ → Q.
2. Direct precipitation route: interface between disordered precipitate and Al matrix → Q’ → Q.

The structures and compositions of precipitates were different between 4M10S and 6016 alloy at the under-aged period, whereas areal density, length and diameter of the precipitates were almost equal. It is suggested that the strengthening in an Al-Mg-Si alloy by Cu addition is given by the Cu related precipitates, which include Cu sub-unit clusters and Q’ sub-unit cells.

## Conclusion

In summary, a combination of atomically-resolved ADF-STEM study and EDS atomic column mapping was carried out to reveal the microstructure and composition of the precipitates at under-aged period in Al-Mg-Si-Cu alloy. We found that β” and Q’ were present at under-aged period as dominant precipitates. Q’ precipitate would probably be formed not only via transformation from Cu sub-unit cluster, but also at the interface between disordered precipitate and Al matrix directly. EDS column mapping showed that Al atoms were replaced with Mg and Si atoms at Mg_1_ columns and Si_3_ columns in β”. The composition of β” at under-aged period were expected as Mg_5-x_Al_2+x_Si_4_ (x ≈ 1). Cu atoms could be present as a solute atom in β”, in which the replacement sites were determined as Al site (Si_3_ column).

## Electronic supplementary material

Supplementary Information

## References

[1] Ardell AJ (1985). Precipitation hardening. Metall. Trans. A.

[2] Ringer SP, Sakurai T, Polmear IJ (1997). Origins of hardening in aged Al-Cu-Mg-(Ag) alloys. Acta Mater..

[3] Polmear IJ (1996). Recent Developments in Light Alloys. Mater. Trans. JIM.

[4] Miao WF, Laughlin DE (1999). Precipitation hardening in aluminum alloy 6022. Scr. Mater..

[5] Myhr OR, Grong O, Andersen SJ (2001). Modelling of the age hardening behaviour of Al-Mg-Si alloys. Acta Mater..

[6] Gupta AK, Lloyd DJ, Court SA (2001). Precipitation hardening in Al-Mg-Si alloys with and without excess Si. Mater. Sci. Eng. A.

[7] Gouma PI, Lloyd DJ, Mills MJ (2001). Precipitation processes in Al-Mg-Cu alloys. Mater. Sci. Eng. A.

[8] Sjölander E, Seifeddine S (2010). The heat treatment of Al-Si-Cu-Mg casting alloys. J. Mater. Process. Technol..

[9] Wang SC, Starink MJ (2005). Precipitates and intermetallic phases in precipitation hardening Al–Cu–Mg–(Li) based alloys. Int. Mater. Rev..

[10] Nurislamova G, Sauvage X, Murashkin M, Islamgaliev R, Valiev R (2008). Nanostructure and related mechanical properties of an Al-Mg-Si alloy processed by severe plastic deformation. Philos. Mag. Lett..

[11] Rajakumar S, Muralidharan C, Balasubramanian V (2011). Predicting tensile strength, hardness and corrosion rate of friction stir welded AA6061-T6 aluminium alloy joints. Mater. Des..

[12] Troeger LP, Starke EA (2000). Microstructural and mechanical characterization of a superplastic 6xxx aluminum alloy. Mater. Sci. Eng. A.

[13] Engler O, Hirsch J (2002). Texture control by thermomechanical processing of AA6xxx Al-Mg-Si sheet alloys for automotive applications - a review. Mater. Sci. Eng. A.

[14] Pogatscher S, Antrekowitsch H, Leitner H, Ebner T, Uggowitzer PJ (2011). Mechanisms controlling the artificial aging of Al-Mg-Si Alloys. Acta Mater..

[15] Xiao Q (2017). Effect of Cu content on precipitation and age-hardening behavior in Al-Mg-Si-xCu alloys. J. Alloys Compd..

[16] Man J, Jing L, Jie SG (2007). The effects of Cu addition on the microstructure and thermal stability of an Al-Mg-Si alloy. J. Alloys Compd..

[17] Ding L, Jia Z, Liu Y, Weng Y, Liu Q (2016). The influence of Cu addition and pre-straining on the natural aging and bake hardening response of Al-Mg-Si alloys. J. Alloys Compd..

[18] Roven HJ, Liu M, Werenskiold JC (2008). Dynamic precipitation during severe plastic deformation of an Al-Mg-Si aluminium alloy. Mater. Sci. Eng. A.

[19] Edwards GA, Stiller K, Dunlop GL, Couper MJ (1998). The precipitation sequence in Al-Mg-Si alloys. Acta Mater..

[20] Ninive PH (2014). Detailed atomistic insight into the β″ phase in Al-Mg-Si alloys. Acta Mater..

[21] Andersen SJ (1998). The crystal structure of the β″ phase in Al-Mg-Si Alloys. Acta Mater..

[22] Vissers R (2007). The crystal structure of the β′ phase in Al-Mg-Si alloys. Acta Mater..

[23] Andersen SJ, Marioara CD, Frøseth A, Vissers R, Zandbergen HW (2005). Crystal structure of the orthorhombic U2-Al4Mg4Si4precipitate in the Al-Mg-Si alloy system and its relation to the β′ and β″ phases. Mater. Sci. Eng. A.

[24] Saito T (2014). HAADF-STEM and DFT investigations of the Zn-containing β” phase in Al-Mg-Si alloys. Acta Mater..

[25] Marioara CD, Andersen SJ, Zandbergen HW, Holmestad R (2005). The influence of alloy composition on precipitates of the Al-Mg-Si system. Metall. Mater. Trans. A.

[26] Andersen SJ, Marioara CD, Vissers R, Frøseth A, Zandbergen HW (2007). The structural relation between precipitates in Al-Mg-Si alloys, the Al-matrix and diamond silicon, with emphasis on the trigonal phase U1-MgAl2Si2. Mater. Sci. Eng. A.

[27] Chen JH, Costan E, Van Huis MA, Xu Q, Zandbergen HW (2006). Atomic pillar-based nanoprecipitates strengthen AlMgSi alloys. Science (80-.)..

[28] Jacobs MH (1972). The structure of the metastable precipitates formed during ageing of an Al-Mg-Si alloy. Philos. Mag..

[29] Matsuda K, Taniguchi S, Kido K, Uetani Y, Ikeno S (2002). Effects of Cu and transition metals on the precipitation behaviors of metastable phases at 523 K in Al-Mg-Si alloys. Mater. Trans..

[30] Saito T, Marioara CD, Andersen SJ, Lefebvre W, Holmestad R (2014). Aberration-corrected HAADF-STEM investigations of precipitate structures in Al-Mg-Si alloys with low Cu additions. Philos. Mag..

[31] Chakrabarti DJ, Laughlin DE (2004). Phase relations and precipitation in Al-Mg-Si alloys with Cu additions. Prog. Mater. Sci..

[32] Li K, Song M, Du Y, Fang X (2012). Effect of minor cu addition on the precipitation sequence of an AS-CAST Al-Mg-Si 6005 alloy. Arch. Metall. Mater..

[33] Ravi C, Wolverton C (2004). First-principles study of crystal structure and stability of Al-Mg-Si-(Cu) precipitates. Acta Mater..

[34] Marioara CD (2007). The effect of Cu on precipitation in Al-Mg-Si alloys. Philos. Mag..

[35] Cayron C, Sagalowicz L, Sagalowicz L, Buffat PA (1999). Structural phase transition in Al-Cu-Mg-Si alloys by transmission electron microscopy study on an Al-4 wt% Cu-1 wt% Mg-Ag alloy reinforced by SiC particles. Philos. Mag. A Phys. Condens. Matter, Struct. Defects Mech. Prop..

[36] Zandbergen HW, Andersen SJ, Jansen J (1997). Structure determination of Mg5Si6:particles in Al by dynamic electron diffraction studies. Science (80-.)..

[37] Hasting Håkon S., Frøseth Anders G., Andersen Sigmund J., Vissers Rene, Walmsley John C., Marioara Calin D., Danoix Frédéric, Lefebvre Williams, Holmestad Randi (2009). Composition of β″ precipitates in Al–Mg–Si alloys by atom probe tomography and first principles calculations. Journal of Applied Physics.

[38] Matsuda K, Ikeno S, Uetani Y, Sato T (2001). Metastable phases in an Al-Mg-Si alloy containing copper. Metall. Mater. Trans. A.

[39] Wolverton C (2001). Crystal structure and stability of complex precipitate phases in Al-Cu-Mg-(Si) and Al-Zn-Mg alloys. Acta Mater..

[40] Wenner S, Jones L, Marioara CD, Holmestad R (2017). Atomic-resolution chemical mapping of ordered precipitates in Al alloys using energy-dispersive X-ray spectroscopy. Micron.

[41] Jones L (2015). Smart Align—a new tool for robust non-rigid registration of scanning microscope data. Adv. Struct. Chem. Imaging.

[42] Ding L (2018). The structural and compositional evolution of precipitates in Al-Mg-Si-Cu alloy. Acta Mater..

[43] Hirsch, J., Skrotzki, B. (Birgit)., Gottstein, G. & Deutsche Gesellschaft für Materialkunde. Aluminium alloys: their physical and mechanical properties. *Mater*. *Sci*. *Forum* **331–337**, 2450 (2008).

