# Supplementary material for: Structural and compositional study of precipitates in under-aged Cu-added Al-Mg-Si alloy
Source: Sci Rep. 2018 Nov 9;8:16629. doi: 10.1038/s41598-018-35134-8 (PMC6226491; doi:10.1038/s41598-018-35134-8)
Supplement: Supplementary file 1 — Supplementary Information [file 41598_2018_35134_MOESM1_ESM.pdf]

Supplementary Information

Structural and compositional study of precipitates in under-aged Cu-added Al-Mg-Si alloy

Takuya Maeda[1*], Kenji Kaneko[1], Takuya Namba[1], Yuki Koshino[1, 2], Yukio Sato[1], Ryo Teranishi[1], and Yasuhiro Aruga[2]

[1] Department of Materials Science and Engineering, Kyushu University, 744, Motooka, Nishi, Fukuoka 819-0395, Japan

[2] Materials Research Laboratory, Kobe Steel, Ltd., 1-5-5, Takatsukadai, Nishi, Kobe 651-2271, Japan

* corresponding author

E-mail: maeda7188@gmail.com

Tel/Fax: +81-92-802-2979

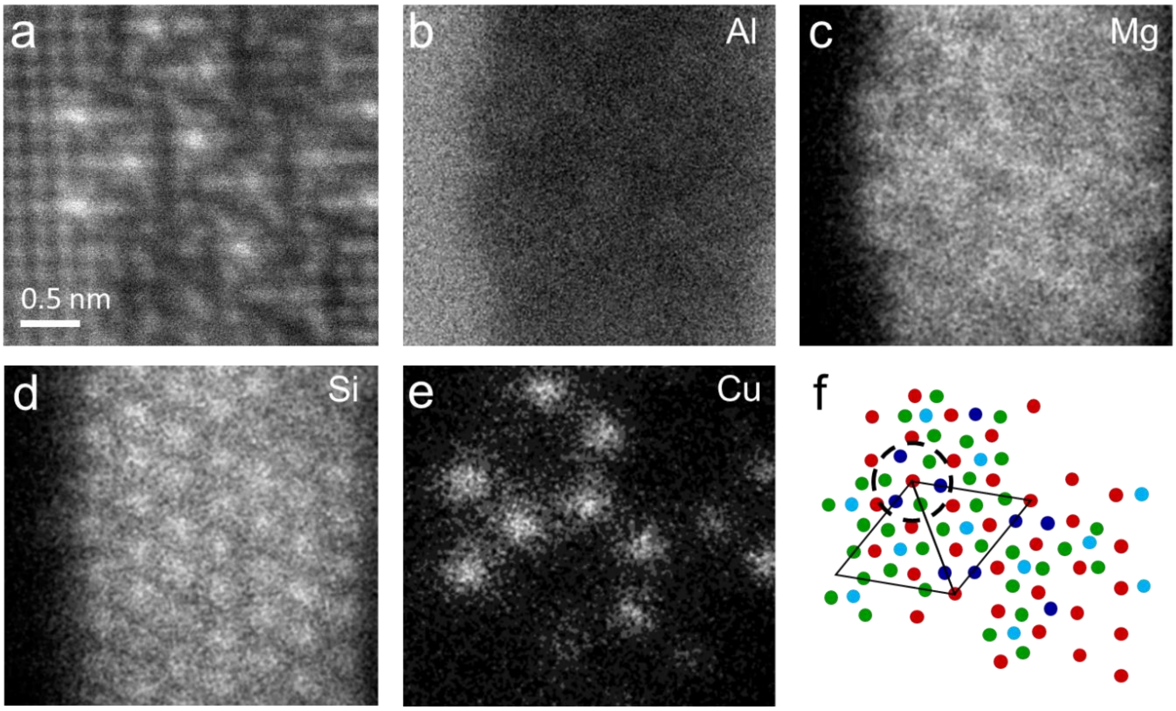

Supplement figure 1. Atomically-resolved HAADF-STEM image and EDS maps of the disordered Q' phase in under-aged 6016 alloy. (a) shows HAADF-STEM image. (b-e) shows chemical map of Al, Mg, Si, and Cu, respectively. (f) shows schematic drawing of proposed Q' structure from the EDS results. Color codes are as follows: blue = Al, green = Mg, red = Si, and Cu = light blue, respectively. Dotted circle indicates three-symmetric Al occupied sites. The composition of Q' can be estimated as $Al_3Mg_9Si_2Cu_7$, as schematically drawn in supplement figure 1(f).
